# An outstanding message of hope: the WHO World Mental Health Report 2022

**DOI:** 10.1017/S2045796022000373

**Published:** 2022-07-14

**Authors:** Benedetto Saraceno, José Miguel Caldas de Almeida

**Affiliations:** Lisbon Institute of Global Mental Health, Lisbon, Portugal

**Keywords:** Mental health, psychiatric hospital, community mental health, ethics

## Abstract

The new WHO World Health Report on Mental Health includes a comprehensive and updated assessment of the current mental health situation at the global level, a critical and well-documented reflexion on the progresses achieved and the failures registered in global mental health, and an indication of the paths and strategies that should be prioritised to ensure the transformations that are urgently needed. The report offers significant enrichments on different areas like social determinants, premature mortality of persons suffering from mental disability, the negative aspects of the persistence of inpatient institutions, the role of people with lived experience as important agents of change, the importance of child and adolescent mental health. The present Editorial stresses the importance of Deinstitutionalisation as a cross-cutting element of all health policy, plans, budgeting and service organisation and draws attention to the fact that the ubiquitous persistence of large psychiatric institutions is a clear indicator that reality is far from declarations despite the UN Convention on the Rights of Persons with Disability.

## Why a new WHO report on mental health?

Twenty years after the World Health Report 2001, *Mental Health: New understanding, new hope* (WHO, 2001), the first authoritative global framework for action in mental health, marking the beginning of a new phase in global mental health, WHO considered the time was come to launch a new World Report dedicated to mental health (WHO, 2022).

The WHO must be heartily congratulated on this excellent and unique contribution to global mental health: a fundamental document that will be a reference for the coming decades. This is what had to be done in this very moment.

Not because the vision and the recommendations of the 2001 Report have lost their validity. But because, in the last 20 years, many aspects of our societies have changed; changes occurred in science and technology, in the recognition of the importance of mental health and in the available responses to mental health care needs. Indeed, it is especially important to policy makers and all other mental health stakeholders to have access to a report from an organisation, with the moral and technical authority as WHO.

This document includes a comprehensive and updated assessment of the current mental health situation at the global level, a critical and well-documented reflexion on the progresses achieved and the failures registered in global mental health, and an indication of the paths and strategies that should be prioritised to ensure the transformations that are urgently needed.

The title of the report – *Transforming mental health for all* – expresses very well what is the main purpose of the report: to provide a support to the global transformation that we need in the present phase of our history.

Most importantly, to attain this objective, *drawing on the latest evidence available, showcasing examples of good practice from around the world, and giving voice to people with lived experience*, the report *highlights why and where change is needed and how it can be achieved on the ground*. (WHO, 2022).

The WHR 2022 presents significant innovations with respect to the WHR 2001. These differences represent an extraordinary improvement both of vision and comprehensiveness of mental health issues. While the Lancet Commission published in 2018 (Patel *et al*., 2018) represented a seminal piece of science providing the state of the art of global mental health, the 2022 WHR represents a seminal, normative and authoritative ‘compass’ for policy makers and professionals.

## Significant enrichments

The report offers eight significant enrichments.

- in previous report, social determinants were considered as essential elements of the aetiological model of mental ill health, while in the present report they are seen as fundamental components of practical intervention to restore mental health of people. In addition, new social determinants are considered, namely, climate change and pandemic.
- the report provides a much more detailed and deep analysis of mental health interventions which go beyond traditional clinical treatment. The report clearly states that interventions to promote and protect mental health should be delivered across multiple sectors because the factors determining mental health are multisectoral in nature.
- the report introduces an especially essential element that was not considered previously, namely the data about premature mortality of persons suffering from mental disability. Policy makers and professionals should consider carefully this dramatic and unrecognised difference between people suffering from mental health disorders and the rest of the population. Such health inequality is not the consequence of any unmodifiable biogenetic characteristic but the results of stigma, discrimination, reduced access to care and severe side effects of uncontrolled psychotropic drugs prescription.
- the report provides more emphasis on the negative aspects of the persistence of inpatient hospitals and institutions which consume significant fraction of mental health budget and human resources.
- at the time of the WHR 2001, the UN Convention of the Rights of Persons with Disability (UN, 2006) was not yet in existence and, quite rightly, the present WHR 2022 stresses the importance of that Convention which for the first time provides a legal frame to the rights of persons with disability. Also, because of the intense public debate which has accompanied the conception, formulation and interpretation of the CRPD, the WHR 2022 stresses the importance, implications and contribution to traditional mental health care provision of the wishes and opinions of people with lived experience: *people with lived experience are important agents of change and can increase awareness and acceptance of mental health conditions among the public* (WHO, 2022).
  *Human rights violations continue to pervade institutions and communities around the world, including health services*. (WHO, 2022). Stressing the fact that too often people with mental health conditions are subject to some of the world's worst human rights abuses by the services responsible for their care, the report presents a comprehensive overview of the measures that may contribute to reduce the use of coercion in mental health services, emphasising the importance of using a mix of strategies designed to modify attitudes, return rights and reshape care environments. Very often new laws and policies are needed to scale up rights; promoting community-based services; strengthening active participation of people with lived experience of mental health conditions; and providing appropriate training for mental health professionals.
  Strongly supporting this view, the report stresses the special importance of a strong investment in the promotion of research contributing to advance the understanding of policies and interventions that are effective in the reduction of coercion, within an implementation science paradigm.
  It is true that there is evidence of the effectiveness of many interventions: among others, staff training, integrated care, shared decision-making interventions (advance care directives, joint care plans and crisis cards), and programmes such as ‘Safewards,’ ‘Six Core Strategies’ and ‘open door policies’ (Barbui *et al*., 2020; Gooding *et al*., 2020). However, much research is still needed to better understand the level of effectiveness each intervention/strategy has in relation to each type of coercion, and what should be done to incorporate these interventions in clinical practice in places with various levels of resources.
  As mentioned in the report the use of involuntary admission and coercive care remains the subject of concern and debate among and between service users and professionals. These conflicting views about involuntary admission remain an important obstacle to the creation of the large consensus that is needed to incorporate a more updated human rights approach in mental health legislations and services across the world. Research alone cannot solve this problem, but in combination with other strategies (particularly service reforms and promotion of debates involving users, professionals and families), certainly it may have a key role in advancing supported decision-making and reducing all forms of coercion.
- Child and adolescent mental health should be considered as a fundamental component both of policy and service provision. Nevertheless, despite the dramatic evidence provided by the age pyramid in most countries of world, still the mental health of children and adolescents do not enough concern the western world (including academic and political international organisations). The WHR 2022 significantly corrects this distortion.
- As mentioned in the report, a profound reorganisation of mental health services is one of the major priorities in mental health and should have two main objectives: *shifting the locus of care for persons with severe mental conditions away from psychiatric hospitals towards the community, and scaling up care for anxiety, depression, and other common mental health conditions by community-based services* (WHO, 2022)
  This model of reorganisation is not new. It has been repeatedly proposed by WHO in the last decades and is now supported by very robust evidence. What is noteworthy in the report is the emphasis given to all questions related to this topic and the clarity with which they are presented. *Discussions on services reform are often confused by a lack of common language. WHO uses the term ‘community-based mental health care’ for any mental health care that is provided outside of a psychiatric hospital. This includes services available through primary health care, specific health programmes (for example HIV clinics), district or regional general hospitals as well as relevant social services. It also includes a range of community mental health services, including community mental health centres and teams, psychosocial rehabilitation programmes and small-scale residential facilities, among others.* (WHO, 2022).
  The way the report discusses the principles of community-based mental health care, describes its different components, and presents examples of how they are implemented in various parts of the globe is exemplary. Reading this document, a policy maker or any other stakeholder can easily understand why community mental health centres or teams and general hospitals are the cornerstone of community-based networks, ensuring the articulations that are needed to scale up care for common mental disorders, provide comprehensive care for persons with severe mental disorders, and develop promotion/preventions activities.
  The reader can also understand, among many other important things, why the reform of mental health services is inseparable of the protection of human rights of persons with mental health conditions, why continuity of care and collaborative care are essential in mental health care, and why even in low-income countries it is important to ensure the existence of some community- based specialised mental health services that may respond to the needs of the more complex cases and support care provided in primary care.
- Implementation has been a major problem in the transformations of mental health systems, services, attitudes and practices that are needed. As the WHO Atlas 2021 shows, only 31% of the mental health plans are fully implemented and only 21% of policies and plans both fully comply with MH rights and are fully implemented, an analogous situation being found in legislation (WHO, 2021). The level of implementation is, as expected, especially low in countries with less financial and human resources, but it affects all countries. Besides additional financial resources in the period of transition from psychiatric hospital-based care, the successful implementation of reforms also requires new policies and reallocation of resources, a good planning, developing new capacities of professionals and establishing new partnerships. Other important requirements, rightly emphasised in the report, are strategies to generate political commitment and establishing a functional mental health unit in the health ministry with an allocated budget and the responsibility for strategic planning. In fact, reforming mental health systems is an overly complex process that requires strong political commitment, and this cannot be obtained without the support of a clear and systematic strategy that is seldom established in most countries. On the other hand, the implementation of all the necessary strategies requires the coordination of a team with technical capacity in different areas, an easy access to the highest levels of decision making in the ministry of health and an allocated budget.

### Final remarks

In conclusion, the authors would like to provide three final remarks:

- The word *deinstitutionalization* appears in the WHR 2022 exclusively in the chapter devoted to transforming mental health care. And all that is said in that chapter about deinstitutionalisation is an important perspective offered by the World Health Organization in this controversial domain: psychiatric institutions should no longer be considered as a component of modern mental health care provision. The authors of the present Editorial applaud this unequivocal approach from WHO.
  However, Deinstitutionalisation is much more than a simple institutional re-engineering (shifting from hospital base care to community care) because it also implies a radical transformation of the overall ‘discourse’ of psychiatry. Deinstitutionalisation has to do with increasing human rights of persons with mental disability, with significant reorientation of financing mental health care, with the need of a radical re-thinking of training of mental health professionals, with a meaningful change of the conception and practice of psychosocial rehabilitation. In other words, Deinstitutionalisation must represent a cross-cutting element of all health policy, plans, budgeting and service organisation.
- If it is understandable that a document issued by the World Health Organization cannot engage in the highly controversial debate about mental health terminology and its obvious important implications, the fact remains that once again this important debate is postponed. Mental illness, mental disorder, mental disability, mental conditions are all different terms (and notions!), and it would be important to overcome existing ambiguities that represent an obstacle to the progress of mental health.
  In present times, the term mental health condition may be the more pragmatic and neutral way to designate all types of mental health problems that can affect human beings. It has the advantage of avoiding the problems and limitations of mental disorders diagnoses, established in accordance with a categorical approach, particularly the ones that result from labelling and ignorance of personal and social circumstances. However, the truth is that a categorical approach continues to be indispensable in clinical work, research and even in some aspects of mental health legislation, policy and services, especially if associated with a dimensional approach. A good example of these hybrid approaches, is the staging approach for mental disorders, proposed in the Lancet Commission (Patel *et al*., 2018), which can contribute to open new avenues in the debate that will be necessary to deepen in the future.
- The understandable optimism of the WHR report which represents the authoritative technical and moral opinion of the United Nations, should be tempered by an honest and severe reality check. In fact, there is a paradoxical divorce between the abundance of declarations and the poverty of results. Indeed, we could say that today mentioning the human rights of users of psychiatric services has become an obligation more dictated by politically correct language than by real projects to defend these rights. According to the WHO Atlas 2020 (WHO, 2021), the percentage of countries indicating full compliance of their mental health policies/plans with human rights instruments has increased notably in all WHO regions since 2014, except in the European Region, where full compliance decreased slightly, from 74% in 2014 to 70% in 2020. Other regions reported a decrease in compliance in 2020 compared with 2017, such as the Africa Region (from 80% to 68% of responding countries) and the Western Pacific Region (from 77 to 67% of responding countries. The modesty of these achievements is troubling but not surprising. Indeed, it should be noted that still 46% of responding countries stated that they allocated 40% of their mental health expenditures to mental hospitals, while 41% of countries allocated more than 60% of their budgets for such facilities. Over 80% of countries reported allocating less than 20% of their total government mental health expenditure to primary health care and mental health prevention and promotion programmes. Similarly, 79% of countries reported allocating no more than 20% to mental health care in general hospitals, and 67% of countries reported allocating no more than 20% to community mental health services.
  These data indicate that in too many countries the organisation of services is still conceived in such a way as not to facilitate the promotion and defence of rights but, on the contrary, to favour their violation (Saraceno, 2022). Indeed, still today countries allocate most of the resources to mental hospitals. According to WHO, *mental hospitals are specialized facilities that provide inpatient care and long-stay residential services for people with mental health conditions, those with severe conditions…They are usually independent and stand-alone.* (WHO, 2021, p. 79).
  However, the stark reality is that in many countries, often those that are least economically developed, people with these disorders continue to reside in large psychiatric hospitals or social care institutions with poor living conditions, inadequate clinical assistance and frequent human rights violations (Caldas de Almeida and Killaspy, 2011). Some authors have shown the risks of human rights violations in mental hospitals (Tansella, 1986; Thornicrof and Bebbington, 1989; Thornicroft and Tansella, 2003; Killaspy, 2006; Thornicroft and Tansella, 2009; Killaspy *et al*., 2018).
  The ubiquitous persistence of large psychiatric institutions is a clear indicator that reality is far from declarations despite the UN Convention on the Rights of Persons with Disability adopted in 2006 by the United Nations General Assembly, signed by 159 states and ratified by 153.The time is ripe for a global mental health advocacy initiative that makes the moral case for the mentally ill. It is unacceptable to continue business as usual. Borrowing from the lessons of our colleagues in other areas of public health, such an initiative could take the form of a Global Alliance for Mental Health, under the umbrella of the World Health Organization, in which mental health professionals work alongside patient, family and public health groups. The practical design of policies, programs and interventions are most likely to be effective when articulated with a moral orientation toward sufferers of mental illnesses. The moral case evoked by Patel, Saraceno and Kleinman in 2006 is still unaddressed (Patel *et al.*, 2006).

## Data Availability

Available.
